# A scoring system based on fusion genes to predict treatment outcomes of the non-acute promyelocytic leukemia pediatric acute myeloid leukemia

**DOI:** 10.3389/fmed.2023.1258038

**Published:** 2023-10-24

**Authors:** Wenwen Weng, Yanfei Chen, Yuwen Wang, Peiting Ying, Xiaoping Guo, Jinfei Ruan, Hua Song, Weiqun Xu, Jingying Zhang, Xiaojun Xu, Yongmin Tang

**Affiliations:** ^1^Division/Center of Hematology-Oncology, Children’s Hospital of Zhejiang University School of Medicine, Hangzhou, China; ^2^The Pediatric Leukemia Diagnostic and Therapeutic Technology Research Center of Zhejiang Province, National Clinical Research Center for Child Health Hangzhou, Hangzhou, China

**Keywords:** acute myeloid leukemia, nomogram, children, fusion gene, prognosis

## Abstract

**Background:**

Fusion genes are considered to be one of the major drivers behind cancer initiation and progression. Meanwhile, non-acute promyelocytic leukemia (APL) pediatric patients with acute myeloid leukemia (AML) in children had limited treatment efficacy. Hence, we developed and validated a simple clinical scoring system for predicting outcomes in non-APL pediatric patients with AML.

**Method:**

A total of 184 non-APL pediatric patients with AML who were admitted to our hospital and an independent dataset (318 patients) from the TARGET database were included. Least absolute shrinkage and selection operation (LASSO) and Cox regression analysis were used to identify prognostic factors. Then, a nomogram score was developed to predict the 1, 3, and 5 years overall survival (OS) based on their clinical characteristics and fusion genes. The accuracy of the nomogram score was determined by calibration curves and receiver operating characteristic (ROC) curves. Additionally, an internal verification cohort was used to assess its applicability.

**Results:**

Based on Cox and LASSO regression analyses, a nomogram score was constructed using clinical characteristics and OS-related fusion genes (*CBFβ::MYH11*, *RUNX1::RUNX1T1*, *KMT2A::ELL*, and *KMT2A::MLLT10*), yielded good calibration and concordance for predicting OS of non-APL pediatric patients with AML. Furthermore, patients with higher scores exhibited worse outcomes. The nomogram score also demonstrated good discrimination and calibration in the whole cohort and internal validation. Furthermore, artificial neural networks demonstrated that this nomogram score exhibits good predictive performance.

**Conclusion:**

Our model based on the fusion gene is a prognostic biomarker for non-APL pediatric patients with AML. The nomogram score can provide personalized prognosis prediction, thereby benefiting clinical decision-making.

## 1. Introduction

Acute myeloid leukemia (AML) is one of the most prevalent types of hematopoietic malignancies, and while there are promising therapies being tested in clinical trials for AML, patient responses remain heterogeneous and the trials lack biomarkers (1). This disease consists of a diverse range of molecular changes; there are only a small number of prototypic genetic lesions in AML patients, and traditional classification is primarily based on morphology (2, 3). Treatment of childhood AML has improved significantly over the past few decades through hematopoietic stem cell transplantation (HSCT), chemotherapy, targeted therapy, optimal risk stratification, and supportive care. However, AML patients treated with the above therapies have unsatisfactory clinical outcomes (4–6). As a result, accurately predicting patients’ prognosis is necessary to improve patient survival.

Fusion genes, as the primary molecular biological anomaly in AML, play a pivotal role in tumor development (7). Currently, fusion genes have been used as molecular markers for leukemia diagnosis, classification, minimal residual disease (MRD) monitoring, risk stratification, and targeted therapy (8). For example, *RUNX1::RUNX1T1* [*t* (8, 21) (q22; q22)] rearrangement, the most frequently observed chromosomal translocation in AML, results in the expression of a transcriptionally repressive fusion protein that functions through the DNA-binding ETO domain (9). *RUNX1::RUNX1T1* made *EGR1* overexpressed to inhibit cell proliferation and promoted apoptosis, which has a relatively favorable outcome in *RUNX1::RUNX1T1*-positive AML (10). Additionally, the fusion gene CBFβ*::MYH11* is caused by central inversion of chromosome 16 inv. (16) (p13.1q16) or *t* (16; 16) (p13.1; q16). The rearrangement disrupts CBF function, leading to blocked myeloid differentiation and ultimately leukemia (11, 12). The connection between fusion genes and the development of AML establishes a theoretical foundation for employing fusion genes in determining clinical outcomes.

In recent years, many studies have developed risk-scoring systems to assess the prognosis of AML. One example is the score established by Schnittger et al. (13), which is based on the expression level of fusion genes. Their findings indicate that fusion transcript levels at diagnosis have a significant impact on the overall survival (OS) and event-free survival (EFS) of AML. Interestingly, the study by Wang (14) showed that the presence of a TEL/AML-1 fusion gene was statistically significant in predicting both OS and EFS, indicating that TEL/AML-1 is a useful biological variable for risk stratification in future clinical trials. Therefore, we believe that the involvement of fusion genes and clinical characteristics in constructing prognostic assessment models could potentially enhance the accuracy of predicting clinical outcomes for AML patients.

In our study, we explored and constructed a scoring nomogram using fusion genes to predict the OS of non-APL pediatric patients with AML. Significantly, we developed a fusion gene-based prognostic signature that showed great potential as a predictor for the prognosis of non-APL pediatric patients with AML. We have highlighted the clinical importance of fusion genes and their potential as promising biomarkers for pediatric non-APL AML patients.

## 2. Materials and methods

### 2.1. Patients and study design

We retrospectively analyzed the clinical data of 184 newly diagnosed non-APL pediatric patients with AML who received continuous treatment at the Children’s Hospital of Zhejiang University School of Medicine (Hangzhou, China) from January 2015 to December 2020 to construct the predictive model. Treatment was performed according to the modified NPCLC-AML97 protocol (15). For patients, induction therapy used either daunorubicin/cytarabine (DA) or daunorubicin/cytarabine/etoposide (DAE). When CR was achieved, the same cycle was repeated for non-APL as consolidation therapy. Intensification therapy consisted of three cycles of intermediate- or high-dose cytarabine (ID/HD Ara-C) every other month for all subtypes of AML. During the intervals of ID/HD Ara-C, DA, homoharringtonine/cytarabine (HA), and etoposide/cytarabine (EA) were given alternatively. Maintenance therapy was given monthly by sequential HA, EA, and doxorubicin/paclitaxel (AT) cycles, and ID/HD Ara-C was administered every 6 months. Central nervous system (CNS) prophylaxis was performed with intrathecal chemotherapy alone. Ara-C, methotrexate (MTX), and dexamethasone (DXM) were administered every 2 weeks during induction, every 2 months during the first year, and every 3 months thereafter. The dosing of MTX, Ara-C, and DXM was different according to the age group: <12 months, MTX 5 mg, Ara-C 12 mg, and DXM 2 mg; up to 23 months, MTX 7.5 mg, Ara-C 15 mg, and DXM 2 mg; up to 35 months, MTX 10 mg, Ara-C 25 mg, and DXM 5 mg; ≥36 months, MTX 12.5 mg, Ara-C 30 mg, and DXM 5 mg. The total course was 2.0 (girls)–2.5 (boys) years from CR. The last date for follow-up was on 31 March 2022, and the median time for follow-up was 2.16 years, ranging from 0.63 to 4.14 years. This study was approved by the Ethics Committee of the Children’s Hospital of Zhejiang University (Approval number: 2022-IRB-130).

An independent dataset containing 318 patients from the TARGET database was used as the target cohort. Case selection criteria for data extraction included patients diagnosed with AML, OS and survival status, WBC, BM blast, PB blast, age, gender, relapse, MRD, mutation gene status, and fusion gene status. For fusion genes and mutation analysis, the bone marrow blood is sent to the company for diagnosis. All treatment regimens adhered to the guidelines stated in the Declaration of Helsinki and received approval from the Institutional Review Board. Informed written consent has been signed by guardians.

### 2.2. Molecular analysis

The French–American–British (FAB) classification system was conducted according to the standard of the World Health Organization (WHO) classification of tumors of the hematopoietic and lymphoid tissues (2020) (16). The diagnosis of fusion genes and mutation genes in bone marrow samples was determined centrally using reverse-transcriptase polymerase chain reaction (RT-PCR), following the standard techniques (17). The process of Ficoll–Hypaque gradient centrifugation was used to concentrate mononuclear cells, which were sent to the company for analysis. The ReverTra Ace qPCR RT kit (Toyobo, Osaka, Japan) was used to reverse transcribe total RNA, which was extracted from leukemic cells at diagnosis. PCR for fusion genes and mutation genes was performed using the primers and procedure according to the published literature (18–21).

### 2.3. Establishment of the predictive nomogram

Prognostic values for clinical characteristics were initially calculated by univariate Cox analysis. We used LASSO-Cox regression models to define risk factors for individual operating systems. A nomogram was created by R (R version 4.0.2) “rms,” “survival” package (22), using clinical characteristics with *p* < 0.05 in the multivariate Cox regression model. Patients were divided into low- and high-risk groups using the optimal cutoff value determined by the X-title method (23). The “rms package” was utilized to create nomograms and calibration plots. Nomogram measurements were performed using calibration curves (24, 25). Calibration plots are used to analyze observed and predicted nomogram probabilities. The R package “survival ROC” was employed to conduct a comparison of accuracy and accuracy predictions using receiver operating characteristic (ROC) curve analysis (26). The accuracy of nomogram survival was assessed by ROC curves (27). Artificial neural network was performed using the “NeuralNetTools” package.

### 2.4. Statistical analyses

The overall survival (OS) was defined as the time period from the date of diagnosis to either the date of death or the last interaction date with the patient. Similarly, event-free survival (EFS) was defined as the time period from the date of first complete remission (CR) until either relapse, death, and second malignancy or the last interaction date with the patient. The differences between the two groups were assessed using the *t*-test, Mann–Whitney U test, or chi-square test. The survival functions were estimated using the Kaplan–Meier method and compared using the log-rank test. All calculations were performed using SPSS 25.0 software (SPSS Inc. Chicago, IL, United States). A significance level of *p* < 0.05 was used, with an evaluation of *p*-values from both sides.

## 3. Results

### 3.1. Clinicopathological and genetic characteristics of non-APL pediatric patients with AML

A total of 184 non-APL pediatric patients with AML from our hospital were collected as a Chinese cohort; of them, 117 were male, accounting for 63.59% of the patients. The median age was 6 years (range: 3–11 years). The median WBC count of these patients was 15.09 × 10^9^/L (range: 6.20 × 10^9^/L–46.79 × 10^9^/L), while the median peripheral blood (PB) blast count was 36% (range: 8%–58%). The levels of bone marrow (BM) blast were 61% (range: 41%–78%). Additionally, the number of patients with M0, M1, M2, M4, M5, M6, and M7 types was 7, 2, 53, 33, 85, 1, and 3, respectively. Table 1 shows the clinical features of non-APL pediatric patients with AML. Additionally, we found that 13 patients (7.07%), 37 patients (20.11%), 11 patients (5.98%), and 6 patients (3.26%) were positive for the fusion genes *CBFβ::MYH11*,*RUNX1::RUNX1T1*, *KMT2A::MLLT3,* and *KMT2A::MLLT10*, respectively. The results of the fusion genes are presented in Table 2. Of all the patients enrolled in this study, 104 cases (56.52%) were of adverse risk and the CR at the end of course 1 was 83.15% (153 cases). Most of the patients (131 cases, 71.20%) had a day 16 measurable residual disease (MRD) <0.1%. There were 136 patients (73.91%) who developed EFS.

**Table 1:** Clinical characteristics of non-APL pediatric patients with AML.

Characteristics	Chinese cohort (*N* = 184)	TARGET cohort (*N* = 318)	Whole cohort (*N* = 502)
**Gender, *n* (%)**			
Male	117 (63.59)	190 (59.75)	307 (61.16)
Female	67 (36.41)	128 (40.25)	195 (38.84)
Age, years, median (range)	6 (3–11)	10 (5–13)	9 (4–13)
WBC count, ×10^9^/L	15.09 (6.2–46.79)	39.35 (13.68–88.25)	26.97 (9.62–76.75)
PB blasts, %, median	36 (8–58)	53.5 (29–76.25)	46.5 (20–69.25)
BM blasts, %	61 (41–78)	74.5 (51–88)	71 (48–86)
**FAB type, *n* (%)**			
M0	7 (3.80)	0 (0)	7 (1.39)
M1	2 (1.09)	59 (18.55)	61 (12.15)
M2	53 (28.80)	95 (29.87)	148 (29.48)
M4	33 (17.93)	95 (29.87)	128 (25.50)
M5	85 (46.20)	49 (15.41)	134 (26.70)
M6	1 (0.54)	7 (2.20)	8 (1.60)
M7	3 (1.63)	13 (4.09)	16 (3.19)

WBC, white blood cell; PB, peripheral blood; BM, bone marrow.

**Table 2:** Status of fusion genes of patients with non-APL pediatric patients with AML.

Characteristics	Level	Chinese cohort (*N* = 184)	TARGET cohort (*N* = 318)	Whole cohort (*N* = 502)
*CBFβ::MYH11*, *n* (%)	Yes	13 (7.07)	63 (19.81)	76 (15.14)
	No	171 (92.93)	255 (80.19)	426 (84.86)
*RUNX1::RUNX1T1*, *n* (%)	Yes	37 (20.11)	71 (22.33)	108 (21.51)
	No	147 (79.89)	247 (78.67)	394 (78.49)
*NUP98::NSD1*, *n* (%)	Yes	2 (1.09)	13 (4.09)	15 (2.99)
	No	182 (98.91)	305 (95.91)	487 (97.01)
*NUP98::KDM5A*, *n* (%)	Yes	1 (0.54)	3 (0.94)	4 (0.80)
	No	183 (99.46)	315 (99.06)	498 (99.20)
*KMT2A::MLLT1*, *n* (%)	Yes	1 (0.54)	2 (0.63)	3 (0.60)
	No	183 (99.46)	316 (99.37)	499 (99.40)
*KMT2A::ELL*, *n* (%)	Yes	2 (1.09)	5 (1.57)	7 (1.40)
	No	182 (98.91)	313 (98.43)	495 (98.60)
*KMT2A::MLLT3*, *n* (%)	Yes	11 (5.98)	13 (4.09)	24 (4.78)
	No	173 (94.02)	305 (95.91)	478 (95.22)
*KMT2A::MLLT10*, *n* (%)	Yes	6 (3.26)	11 (3.45)	17 (3.39)
	No	178 (96.74)	307 (96.55)	485 (96.61)
*DEK::NUP214*, *n* (%)	Yes	2 (1.09)	8 (2.52)	10 (1.99)
	No	182 (98.91)	310 (97.48)	492 (98.01)

*CBFβ::MYH11*, core-binding factor subunit beta-myosin heavy chain 11; *RUNX1::RUNX1T1*, *RUNX1* partner transcriptional co-repressor 1; *NUP98*, nucleoporin 98; *KMT2A::ENL(MLLT1)*, *MLLT1* super elongation complex subunit; *KMT2A::ELL*, elongation factor for RNA polymerase II; *KMT2A::AF9(MLLT3)*, *MLLT3* super elongation complex subunit; *KMT2A::AF10(MLLT10)*, *MLLT10* histone lysine methyltransferase DOT1L cofactor; *DEK::CAN*, *DEK* proto-oncogene.

We downloaded 318 cases of non-APL pediatric patients with AML from the TARGET database. Comparing the clinicopathological and genetic characteristics of the Chinese cohort and TARGET cohort, we found that there were significant differences between the two cohorts in age, WBC count, PB blasts, BM blasts, and FAB types (*p* < 0.001, Table 1). In addition, for fusion genes, we discovered significant differences in *CBFβ::MYH11* (*p* < 0.001, Table 2). Meanwhile, the favorable/intermediate/adverse rates in the TARGET cohort were 52.83% (168/318), 32.39% (103/318), and 14.78% (47/318), respectively, which were significantly different compared with the Chinese cohort (*p* < 0.001, Table 3). However, the Chinese cohort and TARGET cohort demonstrated similar population distributions in CR status at the end of course 1, day 16 MRD, and relapse rate (*p* > 0.05, Table 3).

**Table 3:** Treatment outcome of non-APL pediatric patients with AML.

Characteristics	Level	Chinese cohort (*N* = 184)	TARGET cohort (*N* = 318)	Whole cohort (*N* = 502)
Risk, *n* (%)	Favorable	51 (27.72)	168 (52.83)	219 (43.63)
	Intermediate	29 (15.76)	103 (32.39)	132 (26.30)
	Adverse	104 (56.52)	47 (14.78)	151 (30.07)
CR status at end of course 1, *n* (%)	Yes	153 (83.15)	255 (80.19)	351 (69.92)
	No	31 (16.85)	63 (19.81)	94 (30.08)
Day 16 MRD, *n* (%)	≥0.1%	53 (28.80)	103 (32.39)	156 (31.08)
	<0.1%	131 (71.20)	215 (67.61)	346 (68.92)
EFS, *n* (%)	Yes	136 (73.91)	162 (50.94)	308 (61.36)
	No	48 (26.09)	146 (49.06)	194 (38.64)

MRD, measurable residual disease; CR, complete remission; EFS, event-free survival.

### 3.2. Development of the prognostic signatures for non-APL pediatric patients with AML

**Figure 1:**
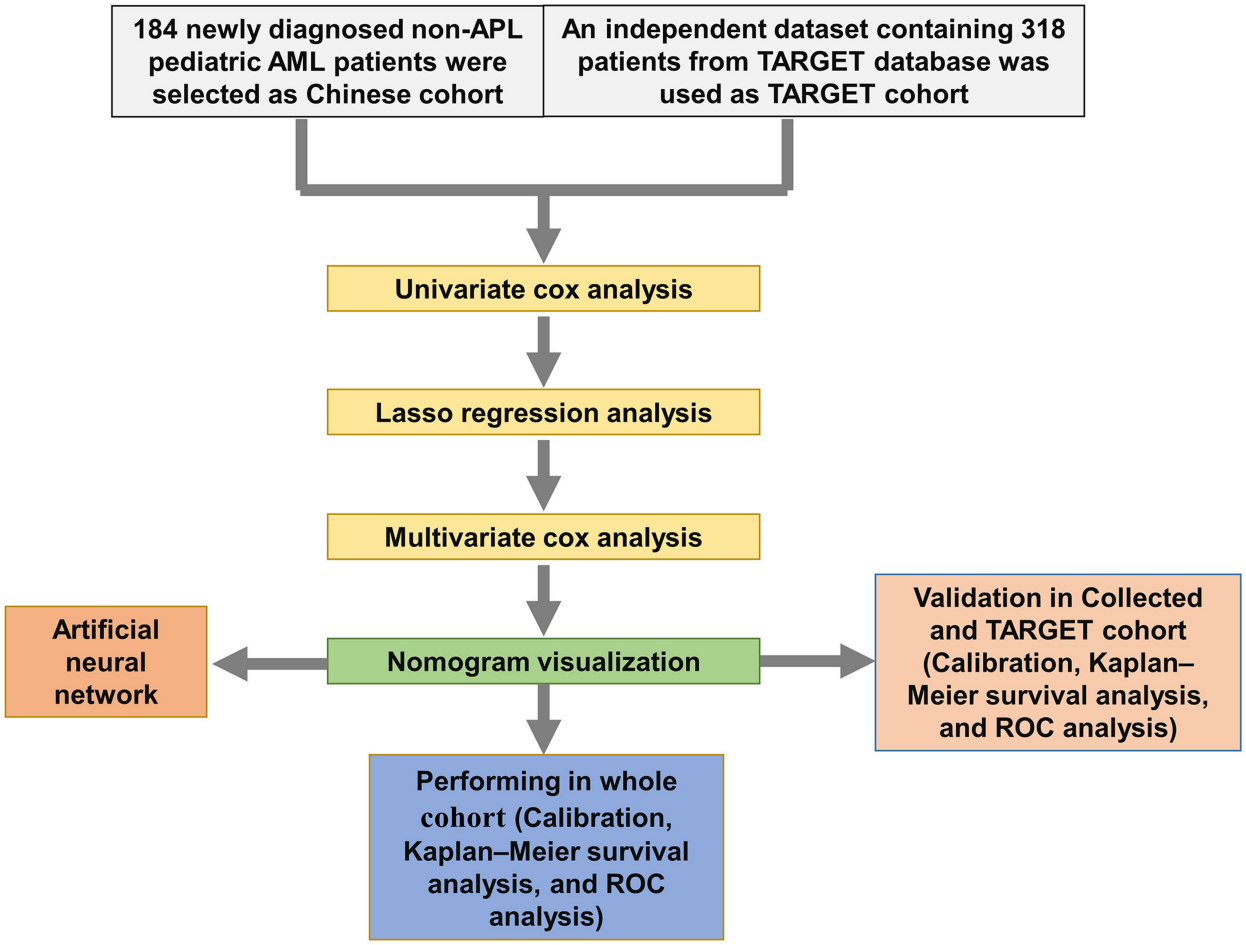
Flowchart of this study.

**Figure 2:**
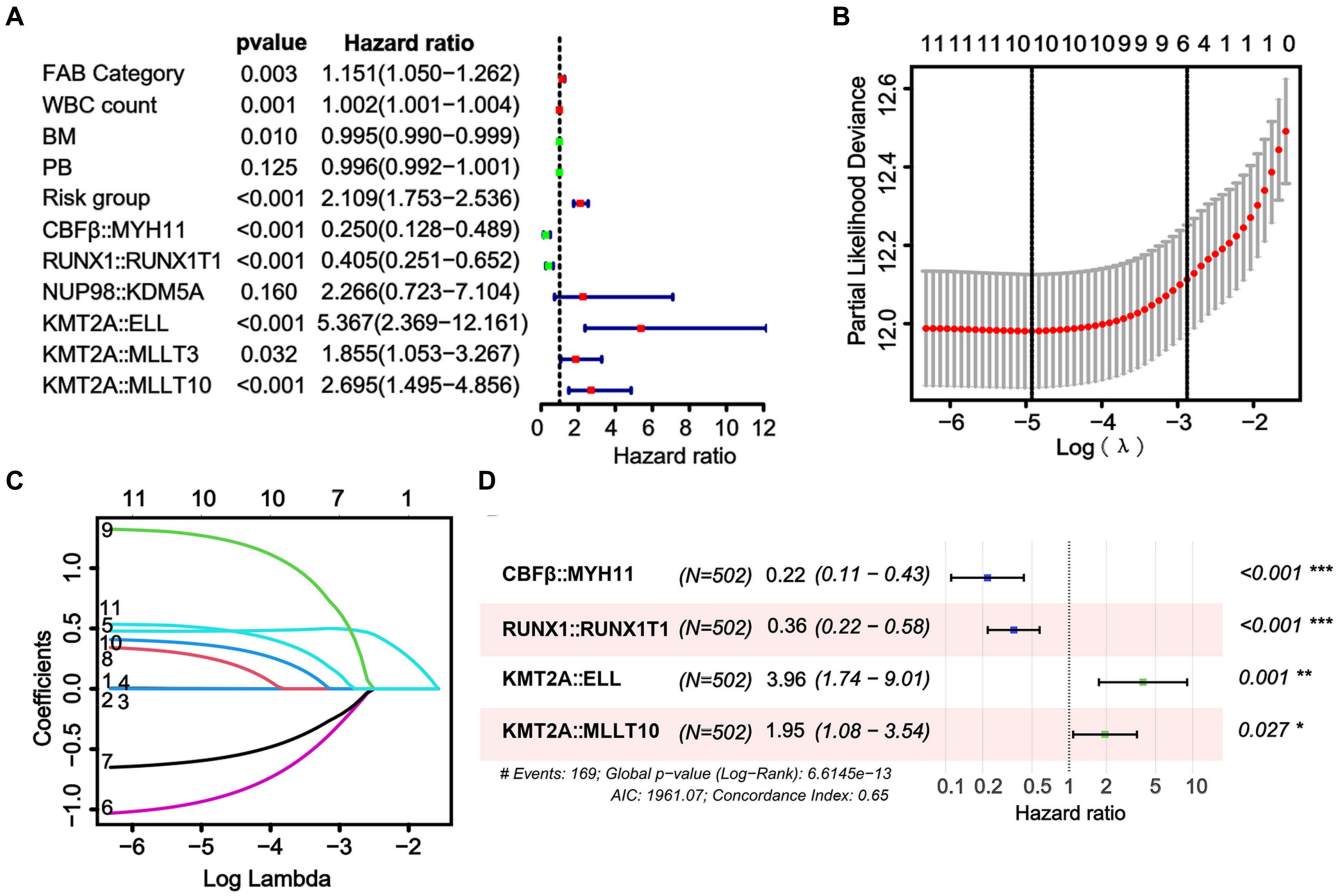
Identification of a prognostic risk model based on fusion genes in the whole cohort. **(A)** Through univariate Cox analysis, the forest plot illustrates the top 11 factors related to survival that were found to be significant. **(B)** The selection of the tuning parameter (lambda) in the LASSO model involved 10-fold cross-validation using the minimum criteria. Dotted vertical lines are drawn at the optimal values determined by the minimum criteria and the standard error of the minimum criteria. **(C)** The LASSO coefficient profiles of the 10 factors related to survival are displayed. **(D)** The results of the multivariate Cox analysis are presented in the forest plot. Six fusion genes were identified as candidates for constructing the risk model in the multivariate Cox analysis.

We screened the prognostic factors for OS in non-APL pediatric patients with AML, and the workflow of the study is presented in Figure 1. By performing univariate Cox regression analysis, survival-related factors were examined from the entire cohort. Using a cutoff point of *p* < 0.2, we identified 11 factors. Among them, 7 potential risk factors (FAB category, WBC count, risk group, *NUP98::KDM5A*, *KMT2A::ELL*, *KMT2A::MLLT3*, and *KMT2A::MLLT10*) and 4 potential protective factors (BM, PB, *CBFβ::MYH11*, and *RUNX1::RUNX1T1*) were included. The survival-related factors were evidenced by the forest plot (Figure 2A).

Using LASSO regression, a predictive model was created from 11 survival-related factors based on the results of the univariate Cox regression. The model selected 10 survival-related factors with non-zero coefficients, following the minimum criteria (Figures 2B,C). Among them, 6 survival-related fusion genes (*CBFβ::MYH11*, *RUNX1::RUNX1T1*, *NUP98::KDM5A*, *KMT2A::ELL*, *KMT2A::MLLT3*, and *KMT2A::MLLT10*) were inspected with multivariate Cox regression analysis. Eventually, based on the minimum Akaike information criterion (AIC) value, a risk model was obtained using four fusion genes (*CBFβ::MYH11*, *RUNX1::RUNX1T1*, *KMT2A::ELL*, and *KMT2A::MLLT10*), of which, *CBFβ::MYH11* and *RUNX::RUNX1T1* were potential protective genes and *KMT2A::ELL*, and *KMT2A::MLLT10* were potential risk genes (Figure 2D). We then obtained risk scores for the fusion genes, which are presented in Table 4.

**Table 4:** Risk score of fusion genes.

Characteristics	Level	Points
*CBFβ::MYH11*	Yes	−1.945
	No	00
*RUNX1::RUNX1T1*	Yes	−1.047
	No	0
*KMT2A::ELL*	Yes	2.049
	No	0
*KMT2A::MLLT3*	Yes	0.856
	No	0

### 3.3. Construction of the nomogram score

**Figure 3:**
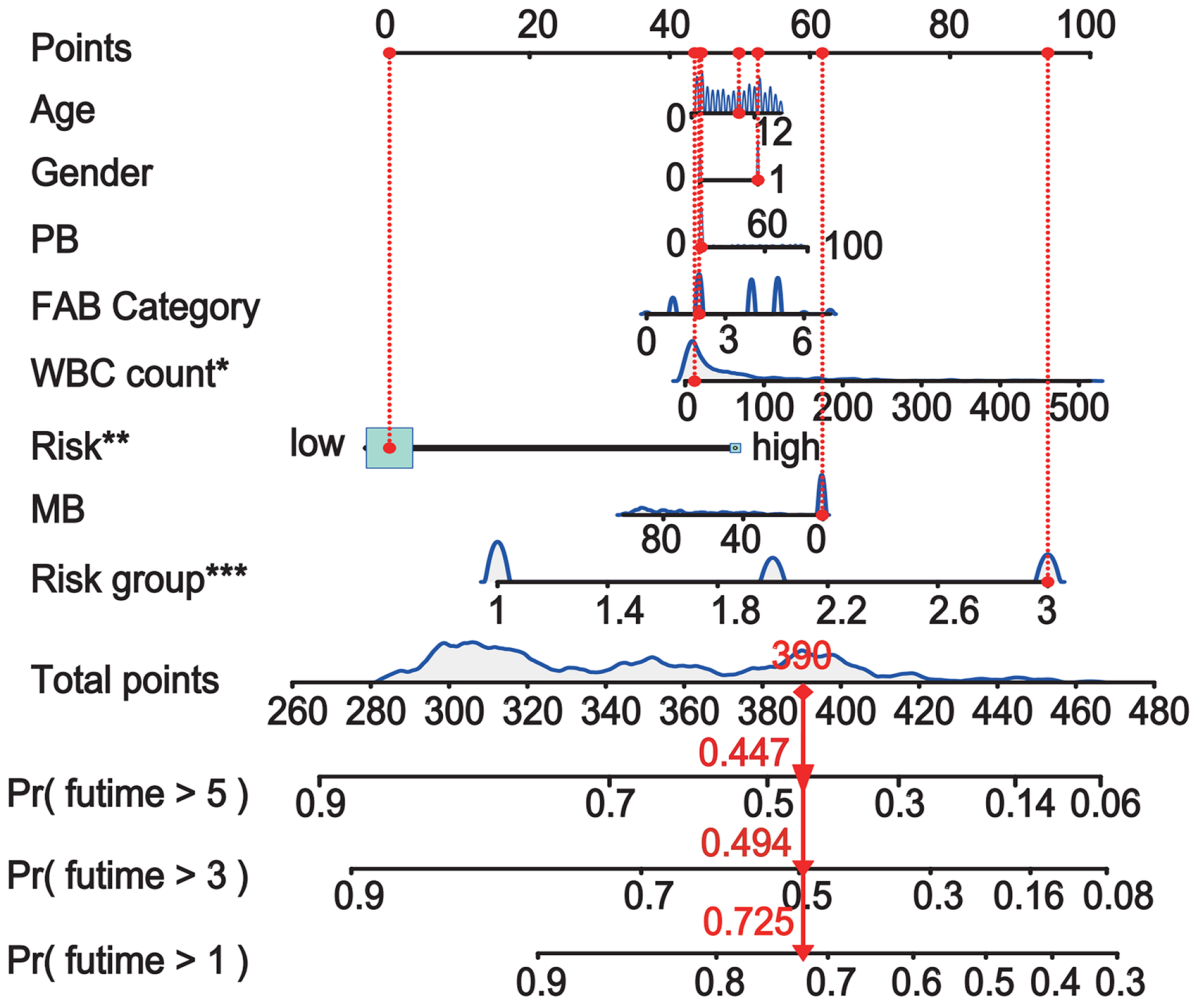
A nomogram is available for predicting the overall survival (OS) of patients at 1, 3, and 5 years.

To enhance the quantitative method used for predicting the prognosis of non-APL pediatric patients with AML, we developed a nomogram score that incorporates age, gender, WBC count, BM blasts, PB blasts, FAB type, risk group, and risk scores of fusion genes. This nomogram has been designed to depict the aforementioned model, with assigned scores given to each term (Figure 3).

### 3.4. Predictive value of the nomogram

**Figure 4:**
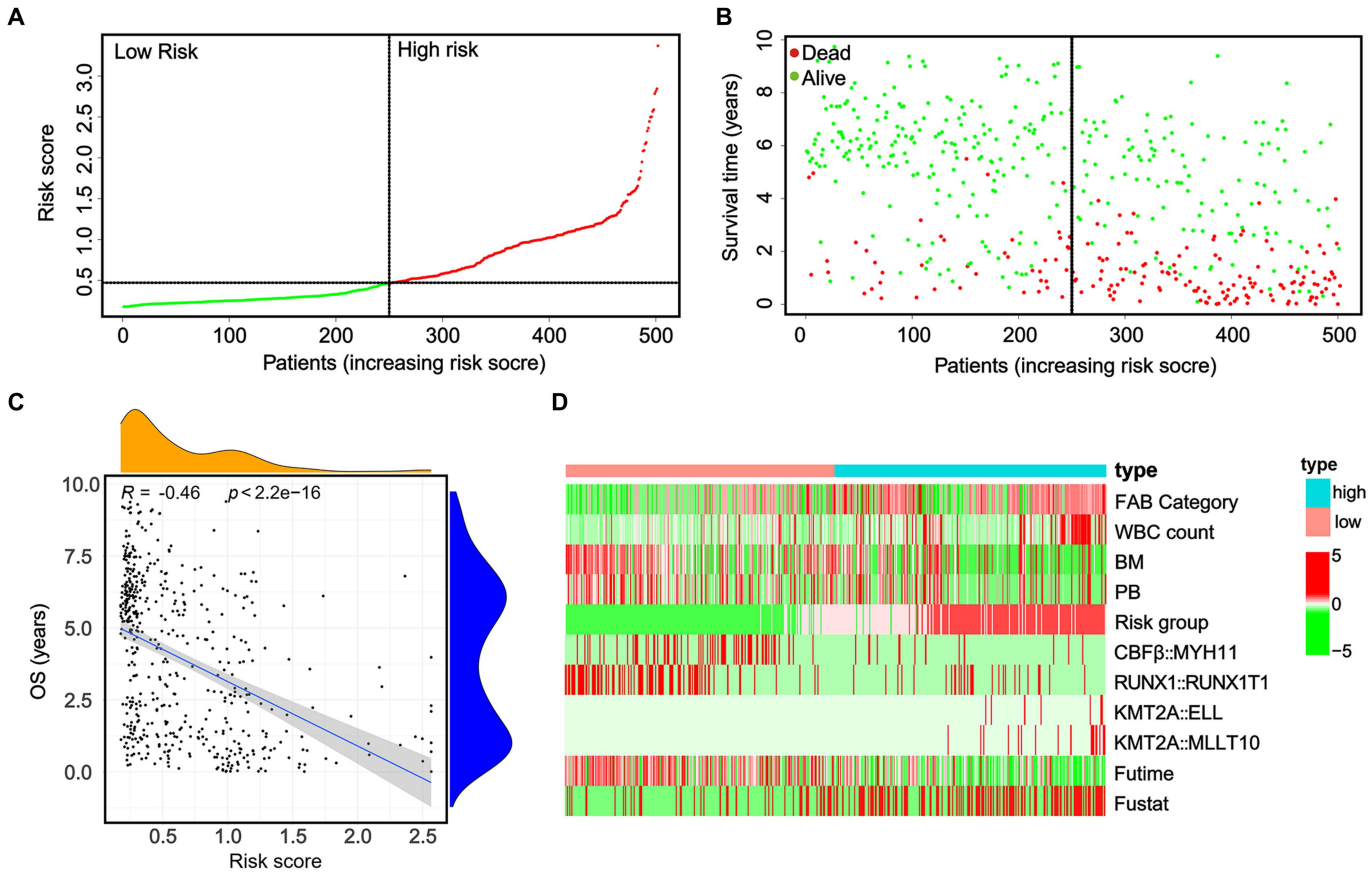
In the whole cohort, the distribution of the nomogram score, patients’ survival status, and fusion gene signature were distributed. **(A)** The patients are divided into two groups equally based on the median risk score threshold, with the low-risk group shown in green and the high-risk group shown in red. **(B)** The survival status of pediatric AML patients without APL in the high-risk and low-risk groups is portrayed, with green representing survival and red representing death. **(C)** The study investigates the association between risk scores and survival times. **(D)** The occurrence of the four fusion genes is illustrated through a heatmap, where the low-risk group is displayed in pink and the high-risk group is displayed in bright blue.

To evaluate the predictive significance of the nomogram, 502 patients were divided into high-risk and low-risk groups based on their individual nomogram scores (threshold: nomogram score = 0.4639), as shown in Figure 4A. Patients with higher risk scores had shorter overall survival and a higher number of deaths (Figures 4B,C). Moreover, higher positive rates (*KMT2A::ELL* and *KMT2A::MLLT10*) and lower positive rates (*CBFβ::MYH11* and *RUNX1::RUNX1T1*) were found in high-risk groups (Figure 4D).

**Figure 5:**
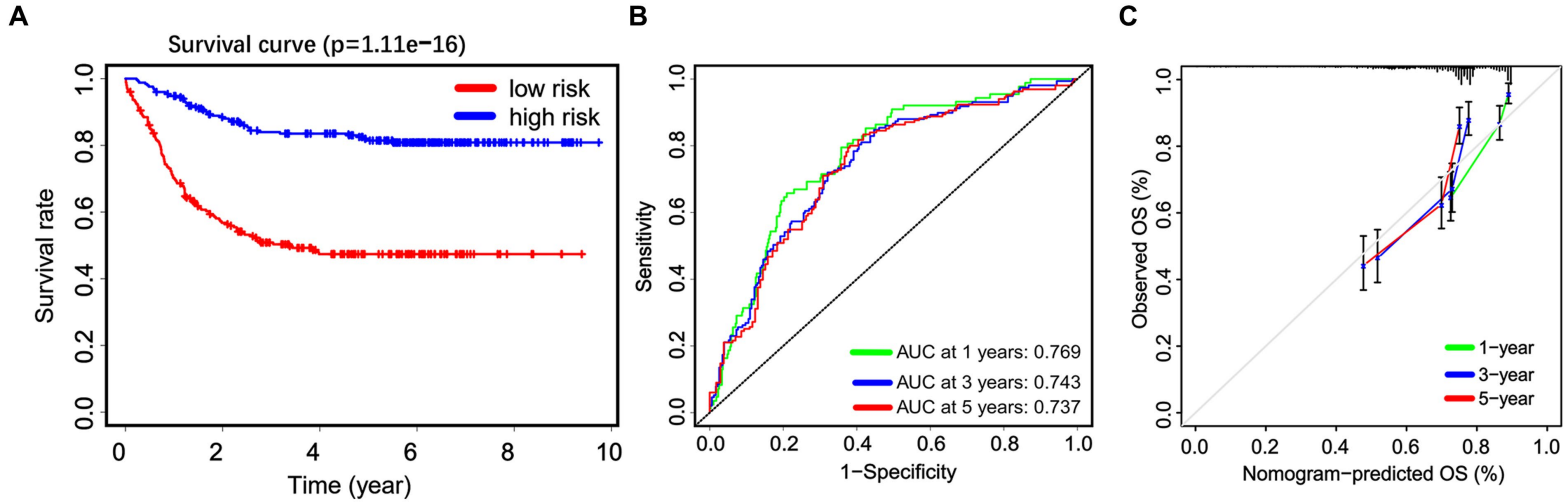
The predictive efficiency of the nomogram. **(A)** Non-APL pediatric patients with AML are divided into high-risk and low-risk groups based on the nomogram score. The Kaplan–Meier plots show the overall survival (OS) of patients in the high-risk and low-risk groups. **(B)** Receiver operating characteristic analysis is utilized to assess the predictive ability of the nomogram score for 1, 3, and 5 years OS in patients. **(C)** Calibration curves are employed to predict the 1, 3, and 5 years OS of patients.

Subsequently, a Kaplan–Meier survival analysis was conducted on the whole cohort, which revealed that patients categorized as high-risk had a significantly worse prognosis (*p* < 0.001; Figure 5A). Furthermore, an ROC analysis was performed to assess the accuracy of this signature in predicting survival rates at 1, 3, and 5 years, resulting in AUC values of 0.769, 0.743, and 0.737, respectively. These findings suggest that this signature demonstrates high sensitivity and specificity (Figure 5B). Additionally, calibration curves were generated to demonstrate a strong agreement between the nomogram model and the actual survival outcomes of patients with non-APL pediatric AML (Figure 5C).

### 3.5. Subgroup-based predictive value of the nomogram

**Figure 6:**
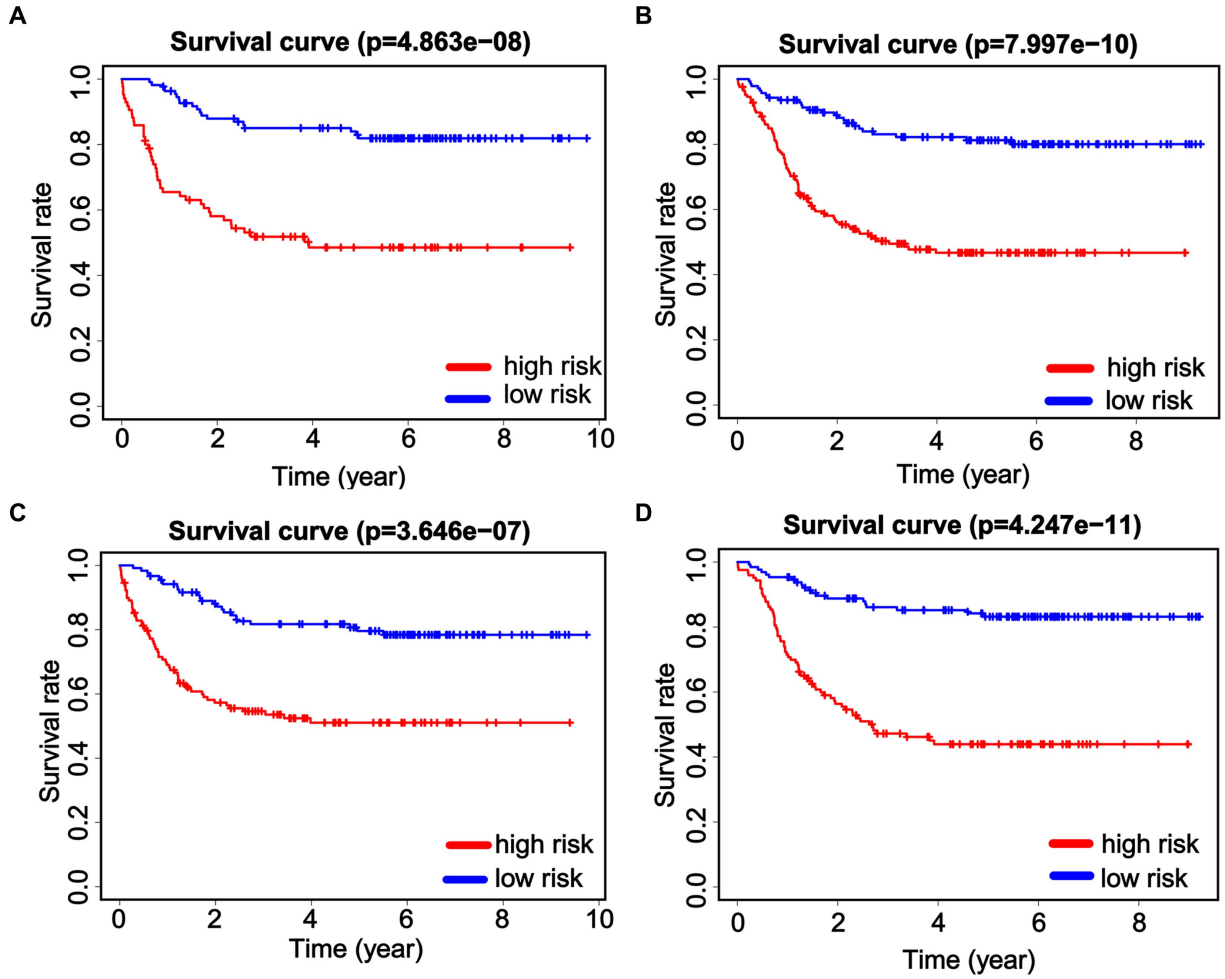
Predictive efficiency of the nomogram score in the subgroups. Kaplan–Meier analysis is performed to evaluate the overall survival (OS) of male patients **(A)** and female patients **(B)**. Identification and verification of the predictive nomogram score in younger (≤8 years, **C**) and older (>8 years, **D**) patients.

To further validate the prognostic value of the nomogram in different subgroups, the patients were grouped according to age and gender. When considering gender subgroups, there were 307 males and 195 females in the whole cohort. Significant differences were observed in the OS between the low-risk and high-risk groups in both the male and female cohorts. Specifically, high-risk patients in both the male and female cohorts exhibited significantly shorter OS (*p* < 0.001, Figures 6A,B). Furthermore, we also found that the patients who were at high risk had notably shorter OS in both the younger group (≤8 years, *N* = 250) and older (>8 years, *N* = 252) patients (*p* < 0.001, Figures 6C,D). These data demonstrate the strong discrimination ability of our nomogram score.

### 3.6. Internal validation of the nomogram

**Figure 7:**
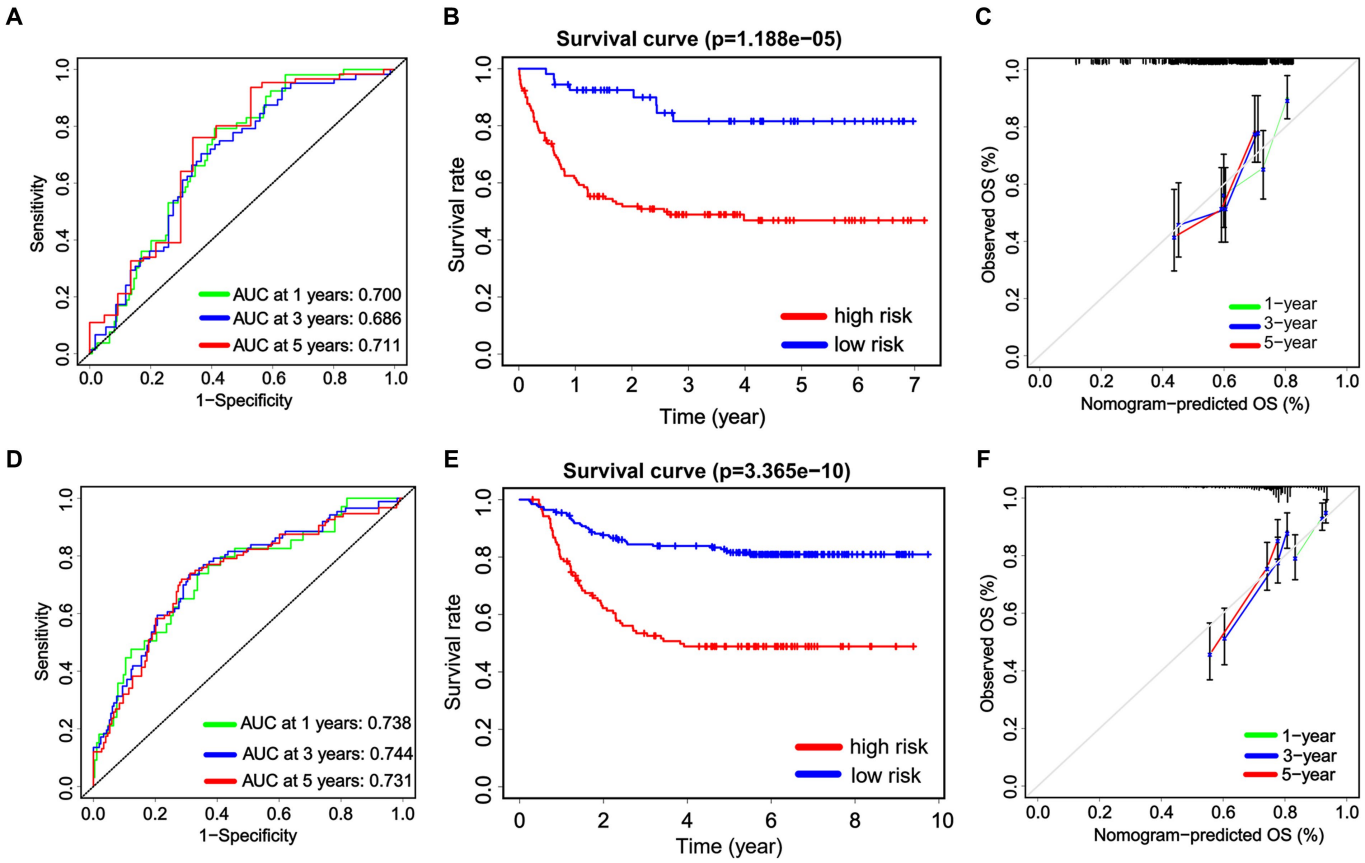
The internal validation of the nomogram score. Receiver operating characteristic analysis for the nomogram score in predicting the patients of 1, 3, and 5 years overall survival (OS) in the Chinese cohort **(A)** and the TARGET cohort **(D)**. The Kaplan–Meier plots are used to present the OS of the patient cohort divided into high-risk and low-risk groups based on the nomogram score in both the Chinese cohort **(B)** and the TARGET cohort **(E)**. Additionally, calibration plots are utilized to predict the 1, 3, and 5 years OS of patients in both the Chinese cohort **(C)** and the TARGET cohort **(F)**.

We internally verified the nomogram score in both the Chinese cohort and the TARGET cohort. In the Chinese cohort, the AUC values for 1, 3, and 5 years intervals were 0.700, 0.686, and 0.711, respectively, indicating a significant level of accuracy (Figure 7A). Furthermore, OS was obtained by plotting a survival curve using the nomogram score scale. The prognosis worsened with an increase in the total score (Figure 7B). In addition, the calibration curves showed that the nomogram closely corresponded to the real-life survival rates of pediatric patients with non-APL AML (Figure 7C). Furthermore, we conducted a comparable analysis procedure in the TARGET cohort, whereby we determined the AUC value for the nomogram score, demonstrating 0.738, 0.744, and 0.731 for 1, 3, and 5 years survival, respectively (Figure 7D). According to the OS curves, patients belonging to the high-signature score group demonstrated considerably poorer overall survival compared with those in the low-signature score group (Figure 7E). Finally, the calibration curve demonstrates that the predicted values aligned with the observed values for the probabilities of 1, 3, and 5 years OS (Figure 7F).

### 3.7. Construction and evaluation of artificial neural network prediction model

**Figure 8:**
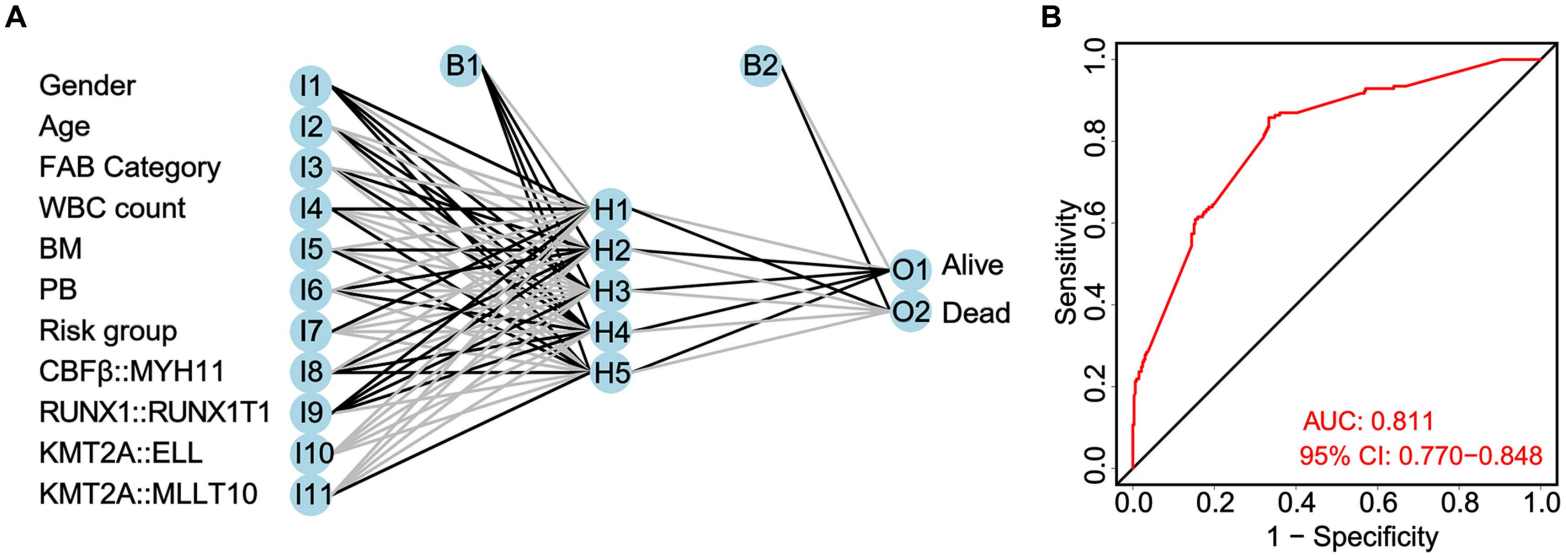
Construction of a neural network prediction model **(A)** and evaluation of the accuracy of the risk score in predicting overall survival (OS) by the ROC curve **(B)**.

Taking clinicopathological characteristics and fusion genes (*CBFβ::MYH11*, *RUNX1::RUNX1T1*, *KMT2A::ELL*, and *KMT2A::MLLT10*) as input variables and OS of non-APL pediatric patients with AML as an output variable, a neural network prediction model is constructed to evaluate the accuracy of risk score prediction for OS. The results demonstrated that the area under the ROC curve predicted by the model for OS was 0.811 (95% CI: 0.770–0.844, Figure 8).

### 3.8. The comparison of three models in prognostic prediction

**Figure 9:**
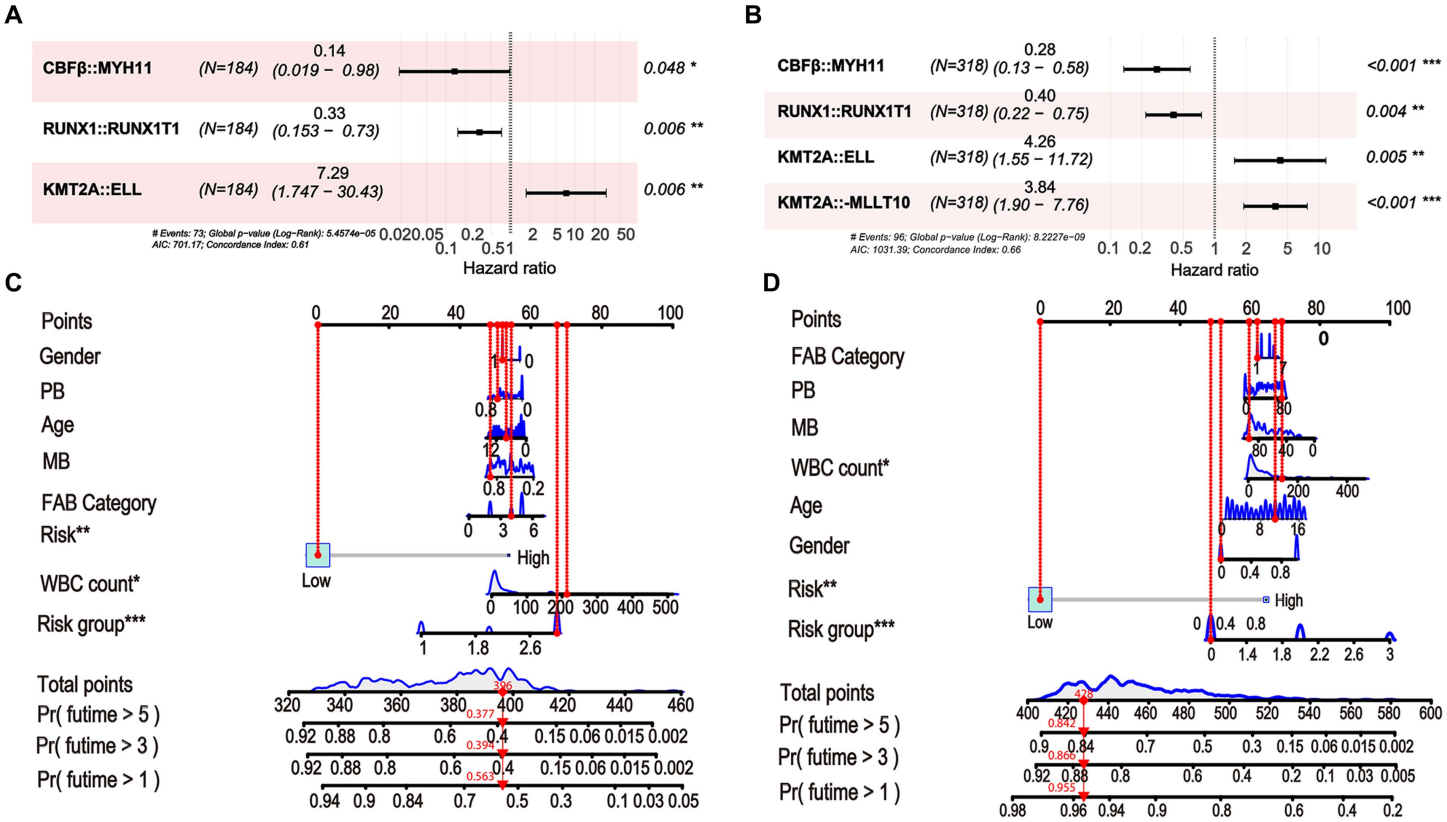
Constructing the nomogram score in the Chinese cohort and the TARGET cohort using fusion genes and clinical characteristics. **(A)** Fusion gene signature selected by multivariate Cox regression in the Chinese cohort. **(B)** Nomogram score for predicting 1, 3, and 5 years OS of non-APL pediatric patients with AML in the Chinese cohort. **(C)** Fusion gene signature selected by multivariate Cox regression in the TARGET cohort. **(D)** Nomogram score for predicting 1, 3, and 5 years OS of non-APL pediatric patients with AML in the TARGET cohort.

**Figure 10:**
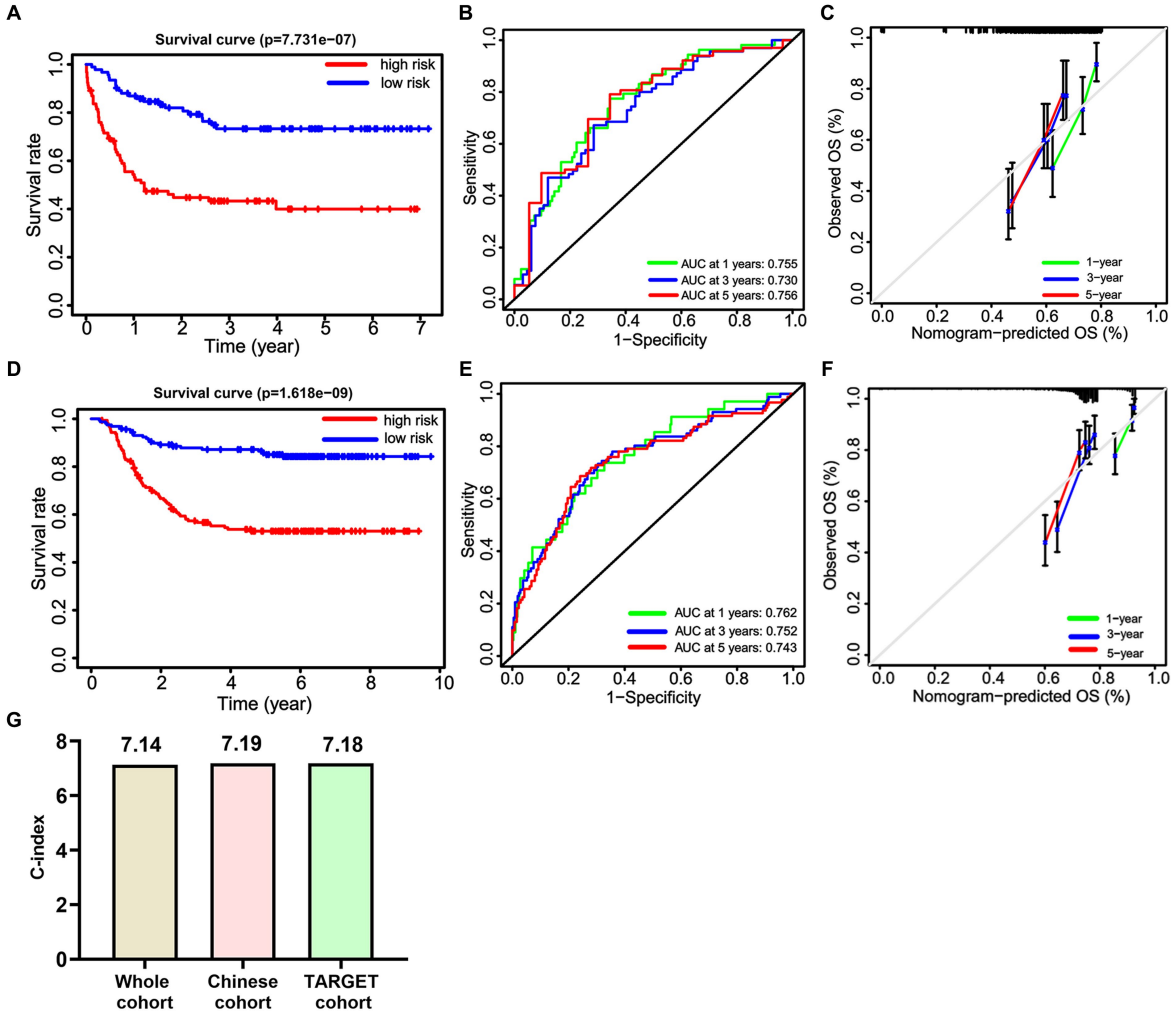
The predictive efficiency of the nomogram score in the Chinese cohort and the TARGET cohort. Kaplan–Meier curves **(A)**, ROC curves **(B)**, and Calibration curves **(C)** of OS in the Chinese cohort. Kaplan–Meier curves **(D)**, ROC curves **(E)**, and Calibration curves **(F)** of OS in the TARGET cohort. **(G)** The C-index of the nomogram score in the whole cohort, the Chinese cohort, and the TARGET cohort.

Here, we compared the differences among the models from the whole cohort, the Chinese cohort, and the TARGET cohort. First, we identified significant variables through univariate Cox analysis, LASSO analysis, and multivariate Cox analysis using the Chinese cohort. These included three OS-related fusion genes (*CBFβ::MYH11*, *RUNX1::RUNX1T1*, and *KMT2A::ELL*). We then built a nomogram score using these fusion genes and clinical characteristics (Figures 9A,B). Furthermore, using the same method as constructing the nomogram score from the whole cohort, we constructed a model using the Chinese cohort. This score included four fusion genes (*CBFβ::MYH11*, *RUNX1::RUNX1T1*, *KMT2A::ELL*, and *KMT2A::MLLT10*), as shown in Figures 9C,D. Interestingly, for the Chinese cohort, Kaplan–Meier analysis displayed that patients in the high-risk group had significantly worse OS than those in the low-risk group (Figure 10A). Meanwhile, the AUC values for 1, 3, and 5 years survival reached 0.755, 0.730, and 0.756, respectively, indicating a significant level of accuracy in the Chinese cohort (Figure 10B). Furthermore, the calibration curve shows that concerning the probabilities of 1, 3, and 5 years OS, the predicted values are consistent with the observed values (Figure 10C). Additionally, we validated the robustness of the nomogram score model in the TARGET cohort. The Kaplan–Meier curve showed that the OS of non-APL pediatric AML patients with a high nomogram score was significantly worse than that of patients with a low nomogram score (Figure 10D). Moreover, the ROC analysis revealed that the AUC values of the nomogram score for 1, 3, and 5 years OS were 0.762, 0.752, and 0.742, respectively (Figure 10E). Calibration curves were conducted to visualize the performance of 1, 3, and 5 years nomograms, indicating that the nomogram performed well (Figure 10F). We calculated the C-index using the restricted mean survival package. The results demonstrated that the C-index derived from the three cohorts was similar to the values of 7.14 in the whole cohort, 7.19 in the Chinese cohort, and 7.18 in the TARGET cohort (Figure 10G).

## 4. Discussion

The treatment of childhood acute myeloid leukemia (AML) has made significant progress in recent decades, but the overall survival rate for children is still below 70%. With continuous improvement in diagnosis and treatment levels, the long-term survival rate of children has improved significantly. However, after the initial remission, the recurrence rate is still 15%–20% (4). In relation to disease progression, recurrence, or death, prognostic biomarkers have the potential to offer valuable insights into the likely outcome of cancer. This knowledge could significantly assist in patient stratification, treatment administration, and disease monitoring within clinical practice, consequently enhancing patient prognosis.

In this study, we retrospectively analyzed the clinical data from 502 patients with non-APL pediatric AML from our hospital and the TARGET database. In the Chinese cohort, the statistical results showed that the prevalence (63.74%) in males is higher than in females, which is in line with the relevant literature (28). In this study, first, we screened out 11 survival-related factors using univariate Cox analysis, most of which are potential risk factors (7 out of 11) that may contribute to the development of AML. Gender and age are the prognostic factors affecting the prognosis of children with AML. Boys have a worse prognosis than girls; additionally, less than 1 year old or more than 10 years old is a poor prognostic factor (29). Although our study has no statistical significance on the correlation analysis of gender, age, and prognosis, considering the small sample size, the statistical analysis of large samples still needs to be further clarified.

Subsequently, we analyzed the factors related to OS in these patients using LASSO regression and multivariate Cox regression. We identified a scoring system based on *CBFβ::MYH11*, *RUNX1::RUNX1T1*, *KMT2A::ELL*, and *KMT2A::MLLT10* that was significantly associated with the OS of patients with non-APL pediatric AML. This is consistent with the findings by Hornung et al. (30), who reported that the *RUNX1::RUNX1T1* fusion is associated with a more favorable outcome compared with AML patients without fusion. In addition, Kadkol et al. (31) found that patients who express *CBFβ-MYH11* fusion transcripts respond favorably to high-dose chemotherapy and are generally spared allogeneic bone marrow transplantation. These previous studies provide theoretical support for our use of *CBFβ::MYH11* and *RUNX1::RUNX1T1* for prognostic evaluation of OS. However, Ishikawa pointed out that the positivity of *CBFβ::MYH11* and the positivity of *RUNX1::RUNX1T1* were associated with poorer prognosis (32). Therefore, it is necessary to conduct in-depth mechanism exploration in future to help reveal the role of *CBFβ::MYH11* and *RUNX1::RUNX1T1* in AML progression and their association with prognosis. Recently, the findings by Zhang et al. (33) inferred that the concurrent AML might be attributed to the fusion mutation in the *KMT2A::ELL* gene, which further enhances the credibility of our results. Although the duplication of KMT2A is a rare molecular event in childhood leukemia, multiple case reports showed that patients with *KMT2A::MLLT10* duplication remained in first complete remission with negative minimal residual disease at 3.5 years from diagnosis (34, 35). Constructing prognostic models with this cryptic translocation may shed more light on the management of AML.

Nomograms can provide more accurate predictions by being simple, easy to understand, and easy to use in clinical procedures (36, 37). There is limited data available on the nomogram for predicting the prognosis of pediatric acute myeloid leukemia patients using fusion genes and clinical factors. Therefore, we generated a nomogram that incorporates fusion genes and clinical characteristics as prognostic indicators, revealing the factors that affect survival. Kaplan–Meier survival analysis, ROC curve, and calibration curve were performed, and the results showed that the risk score of the nomograms could effectively predict OS of patients with non-APL pediatric patients with AML. This research provided the first evidence that the nomogram score, which included fusion genes and other clinical information, is a promising independent predictor of survival. To assess the generalizability of the nomogram score, we extended the nomogram score to various subgroups, and those nomogram scores still demonstrated high predictive power among populations of different genders and ages. Furthermore, we conducted validation in the Chinese cohort and the TARGET cohort, further confirming the accuracy of the model. The result of internal validation provided evidence supporting the usefulness of the nomogram score. The validation results show that the model has outstanding predictive ability for prognosis. Additionally, the findings from the artificial neural network analysis supported the results obtained through other analyses, further emphasizing the value of this scoring system in evaluating the prognosis of non-APL pediatric AML patients.

Using the method of generating a nomogram score for the whole cohort, we generated new nomogram scores for the Chinese cohort and the TARGET cohort, respectively. Interestingly, although the fusion genes involved were slightly different, the predictive abilities of the nomogram scores established by the Chinese cohort were similar to those of the nomogram scores established by the TARGET cohort, confirmed by Kaplan–Meier curves, ROC curves, and calibration curves. However, Liu et al. (38) found a substantially distinct genomic alteration profile by comprehensively comparing the Chinese cohort with the western AML cohort. In addition, this distinct driver profile is clinically relevant. Unlike the RNA-seq analysis in the study by Liu et al. (38), in this study, we analyzed nine fusion genes that co-exist in both the Chinese cohort and the TARGET cohort. These nine fusion genes are relatively common and are associated with prognosis. Therefore, the fusion genes screened in the Chinese cohort (*CBFβ::MYH11*, *RUNX1::RUNX1T1,* and *KMT2A::ELL*) are similar to those screened in the TARGET cohort (*CBFβ::MYH11*, *RUNX1::RUNX1T1*, *KMT2A::ELL*, and *KMT2A::MLLT10*). Due to the limited number of fusion genes included, the predictive ability of the model constructed by the Chinese cohort was similar to that of the model constructed by the TARGET cohort. Our findings not only demonstrate the high universality of nomogram scores generated using fusion genes but also expand the knowledge of fusion genes in the prognostic evaluation of AML patients. Moreover, these findings also prove that the nomogram score we generated is credible. Although the prognostic evaluation of these four fusion genes is widely known, our research, for the first time, integrated four fusion genes and incorporated clinical data to predict patients’ overall survival, which provides more data support for the use of fusion genes in prognostic assessment. Moreover, obtaining more fusion genes and mutation genes from AML patients through RNA-seq analysis will be more helpful in building more accurate predictive models for OS.

AML is a highly aggressive hematological malignancy with a poor prognosis. Therefore, prognostic prediction may contribute to better therapeutic response. Growing evidence has proposed the notion that fusion genes provide new insights into the mechanisms of molecular events implicated in AML. To the best of our knowledge, we first provide clear evidence supporting fusion genes in prognostic analysis for non-APL pediatric patients with AML using published data and the Chinese cohort, which provides a foundation for the development of future treatment strategies and targeted therapies for AML. Furthermore, our study provides a new model based on fusion genes for prognostic analysis of AML, which helps guide the development of treatment strategies and the implementation of personalized treatment. Notably, the strong predictive power of our model confirms the significance of fusion genes as vital factors in the prognosis analysis of AML. This serves as a foundation for integrating additional fusion genes into the prognosis analysis of AML.

However, there are several shortcomings in this study. First, we developed a nomogram score model to predict OS in non-APL pediatric patients with AML. However, this model has not been explored for predicting patient relapse and CR and MRD levels. Second, the mutation gene status is also not included, for example, *FLT3-ITD*, *WT1*, *TET2*, *TP53*, and *CEBPA*, which is highly related to chemotherapy resistance and clinical outcomes. Although our study has limitations, it still provides a reference value to adjust management strategies for the high-risk prevention and control of AML patients.

## 5. Conclusion

In conclusion, using the Chinese cohort and the TARGET cohort, we have established a nomogram scoring system based on fusion genes and clinical characteristics, which showed sufficient predictive power for the OS of non-APL pediatric patients with AML. Importantly, our data showed that this nomogram scoring system has sufficient predictive power. This scoring system could provide clinicians with useful information for managing measures and making individualized risk predictions of clinical events. This nomogram score helps identify patients with a poor prognosis and improve treatment strategies.

## Data availability statement

The original contributions presented in the study are included in the article/supplementary material, further inquiries can be directed to the corresponding authors.

## Ethics statement

The studies involving humans were approved by the Ethics Committee of the Children’s Hospital of Zhejiang University (Approval number: 2022-IRB-130). The studies were conducted in accordance with the local legislation and institutional requirements. Written informed consent for participation in this study was provided by the participants’ legal guardians/next of kin.

## Author contributions

WW: Conceptualization, Data curation, Investigation, Resources, Supervision, Validation, Visualization, Writing – original draft. YC: Data curation, Investigation, Resources, Supervision, Validation, Visualization, Writing – original draft. YW: Data curation, Investigation, Resources, Supervision, Validation, Visualization, Writing – original draft. PY: Data curation, Investigation, Resources, Software, Validation, Visualization, Writing – original draft. XG: Data curation, Investigation, Resources, Validation, Visualization, Writing – original draft. JR: Data curation, Investigation, Resources, Validation, Visualization, Writing – original draft. HS: Data curation, Investigation, Methodology, Validation, Visualization, Writing – original draft. WX: Conceptualization, Investigation, Validation, Visualization, Writing – original draft. JZ: Data curation, Investigation, Visualization, Writing – original draft. XX: Conceptualization, Investigation, Methodology, Project administration, Resources, Supervision, Validation, Visualization, Writing – review & editing. YT: Conceptualization, Formal analysis, Funding acquisition, Investigation, Methodology, Project administration, Resources, Supervision, Validation, Visualization, Writing – review & editing.
